# Fractional Anisotropy in Selected, Motor-Related White Matter Tracts and Its Cross-Sectional and Longitudinal Associations With Motor Function in Healthy Older Adults

**DOI:** 10.3389/fnhum.2021.621263

**Published:** 2021-06-22

**Authors:** Jessica Oschwald, Susan Mérillat, Lutz Jäncke, Rachael D. Seidler

**Affiliations:** ^1^University Research Priority Program “Dynamics of Healthy Aging”, University of Zurich, Zurich, Switzerland; ^2^Department of Neuropsychology, Psychological Institute, University of Zurich, Zurich, Switzerland; ^3^Department of Applied Physiology and Kinesiology, University of Florida, Gainesville, FL, United States

**Keywords:** white matter microstructure, motor function, longitudinal, correlated change, healthy aging, fractional anisotropy, structural equation modeling (SEM), latent growth curve model (LGCM)

## Abstract

**Background:**

While it is well-known that deficits in motor performance and brain structural connectivity occur in the course of healthy aging, it is still unclear if and how these changes are related to each other. While some cross-sectional studies suggest that white matter (WM) microstructure is positively associated with motor function in healthy older adults, more evidence is needed. Moreover, longitudinal data is required to estimate whether similar associations can be found between trajectories of change in WM microstructure and motor function. The current study addresses this gap by investigating age-associations and longitudinal changes in WM microstructure and motor function, and the cross-sectional (level-level) and longitudinal (level-change, change-change) association between these two domains.

**Method:**

We used multiple-occasion data (covering 4 years) from a large sample (*N* = 231) of healthy older adults from the Longitudinal Healthy Aging Brain (LHAB) database. To measure WM microstructure, we used diffusion-weighted imaging data to compute mean FA in three selected WM tracts [forceps minor (FMIN); superior longitudinal fasciculus (SLF); corticospinal tract (CST)]. Motor function was measured via two motor speed tests (grooved pegboard, finger tapping) and one motor strength test (grip force test), separately for the left and the right hand. The statistical analysis was conducted with longitudinal growth curve models in the structural equation modeling framework.

**Results:**

The results revealed longitudinal decline and negative cross-sectional age-associations for mean WM FA in the FMIN and SLF, and for motor function in all tests, with a higher vulnerability for left than right hand motor performance. Regarding cross-domain associations, we found a significant positive level-level correlation among mean WM FA in the FMIN with motor speed. Mean FA in SLF and CST was not correlated with motor performance measures, and none of the level-change or change-change associations were significant. Overall, our results (a) provide important insights into aging-related changes of fine motor abilities and FA in selected white matter tracts associated with motor control, (b) support previous cross-sectional work showing that neural control of movement in older adults also involves brain structures outside the core motor system and (c) align with the idea that, in healthy aging, compensatory mechanisms may be in place and longer time delays may be needed to reveal level-change or change-change associations.

## Introduction

Life expectancy has risen steadily due to innovations in medicine and improved living standards. In 2015, life expectancy at birth exceeded 80 years in 22 European countries (World Health Organization, [WHO], 2016). Globally, it is estimated to increase by a further 6 years until 2050 (United Nations, 2017). Understanding how the central nervous system changes with age contribute to declines in function is critically important for enhancing productivity and quality of life for this aging population. It is well known that aging is associated with degeneration of the central nervous system and decreases in motor performance (Seidler et al., 2010). To date, however, work in this area has been largely cross-sectional and more focused on regional measures of brain structure and function rather than network connectivity (Oschwald et al., 2019a). The brain’s network structure underlies neural communication and functional activity; thus, studying how it changes over time may provide key insights into age-related functional declines.

Diffusion-weighted MRI (DW-MRI) allows investigation of structural integrity of the brain’s white matter (WM) connectivity pathways. This technique is sensitive to diffusion of water molecules, which is spatially bounded by large WM tracts in the brain. Cross-sectional DW-MRI studies generally report lower fractional anisotropy (FA) in older individuals (reviewed in Oschwald et al., 2019a). A few longitudinal DW-MRI studies have been conducted; similar to what has been reported with other imaging modalities, prefrontal WM exhibits accelerated declines relative to other areas of the brain (cf. Barrick et al., 2010; Sullivan et al., 2010; Teipel et al., 2010) while sensorimotor WM exhibits less change (de Groot et al., 2016). In contrast, a recent, large (*n* > 900) cross-sectional study challenges the notion that sensorimotor regions exhibit reduced aging effects relative to more anterior prefrontal cortex; Taubert et al. (2020) found disproportionately reduced brain volume, iron, and myelin in the pre- and postcentral gyri in older individuals. This study did not evaluate WM tracts, however, leaving open the questions of how sensorimotor WM tracts change over time and whether such changes are correlated with motor function.

Whether or not the sensorimotor fibers are spared with age, there is certainly evidence of age effects on motor function. Gait and balance (Studenski et al., 2011), grip force (Bohannon, 2008), and other activities in everyday life decline with age and impact quality of life. Interestingly, performance of these behaviors is associated with prefrontal activity (Heuninckx et al., 2008; Seidler et al., 2010; Carson, 2018) as well as with prefrontal (Verlinden et al., 2016; Moscufo et al., 2018; Massa et al., 2019) and corpus callosum (Fling et al., 2011; Fling and Seidler, 2012). WM integrity in older adults, potentially reflecting compensation (Heuninckx et al., 2008). However, more evidence on the association between WM connectivity and motor function in healthy older adults is needed. Importantly, longitudinal data are required to more precisely delineate the trajectories of decline and to better understand if the associations between WM microstructure and motor behavior, and particularly associations of their changes, are reflective of compensation, maintenance, or other patterns in healthy older adults (Zahodne and Reuter-Lorenz, 2019).

In the current study, we leverage data from the Longitudinal Healthy Aging Brain (LHAB) database to evaluate cross-domain associations between brain WM microstructure and measures of manual motor function (motor strength: grip force; motor speed: grooved pegboard test and tapping speed) in healthy older adults. The LHAB database project is currently conducted at the University Research Priority Program (URPP) “Dynamics of Healthy Aging” of the University of Zurich (Zöllig et al., 2011). Our analyses include data that were acquired at four time points spanning over 4 years.

We used latent growth curve models (LGC) estimated in the structural equation modeling framework (SEM) to examine change in WM microstructure and motor function, as well as cross-sectional, and longitudinal associations among the two domains.

LGC is a statistical technique for the analysis of longitudinal data (McArdle and Epstein, 1987; McArdle, 2009; for a tutorial see Ghisletta and McArdle, 2012). LGC models estimate longitudinal growth processes as latent (i.e., unobserved) variables, including a latent intercept which reflects the initial level of a variable of interest (e.g., WM microstructural properties or motor performance at baseline) and a latent slope, which reflects the rate of change in this variable over time. An advantage of LGC models over traditional regression models is that besides such average (i.e., fixed) effects, they can capture interindividual variances (i.e., random effects) in intraindividual change. Of specific interest in the present study, two univariate LGCs (i.e., LGC that estimate the growth process in one variable) can be combined into a bivariate LGC to model parallel change processes, including cross-domain associations between baseline levels of two variables (level-level), baseline level in one variable and changes in the other (level-change) and between two change processes (change-change) (Oschwald et al., 2019a). Importantly, advanced statistical techniques such as LGC are required to appropriately estimate within-person change (King et al., 2018), and to disentangle the complex longitudinal associations between changes in multiple variables – both questions of pivotal importance in the field of aging neuroscience.

Cross-sectional age-associations and longitudinal decline was estimated in the forceps minor (FMIN), the superior longitudinal fasciculus (SLF), and the corticospinal tract (CST). The CST is the main motor control projection tract. It plays a critical role in fine motor control of hand and finger movements (cf. Isa, 2012) such as those required for the tasks studied here. The FMIN connects the two prefrontal cortices via the anterior corpus callosum, whereas the SLF connects prefrontal cortex largely with parietal regions. While all three tracts have been implicated in motor function (Farbota et al., 2012; Henley et al., 2014; Wang et al., 2016; Reid et al., 2017; Giacosa et al., 2019; Maltais et al., 2020), the SLF and FMIN largely support executive functions such as attention and working memory (Mamiya et al., 2018; Nakajima et al., 2020) and do not have direct projections to spinal motor neurons. Thus, here, we refer to the CST as belonging to the motor system, and FMIN and SLF as being outside of the motor system. To measure WM microstructure, we chose to follow a region-of-interest (ROI) based approach in the present study, averaging WM FA across the selected fiber tracts, since the extraction of ROIs (as opposed to voxel-wise estimates) enabled us to estimate parallel change in the SEM framework. We used FA as an index of WM microstructure as it is a comparatively well-researched metric and provides a general estimate of the change in the WM fiber organization (see Jones et al., 2013, for considerations on the interpretation of FA).

Age-associations and change in motor performance was assessed with finger tapping, pegboard performance and grip force given that performance in these tests has been previously shown to decline with age. Based on the findings of de Groot et al. (2016), we hypothesized that the FMIN and SLF would exhibit greater change over time than the CST, and would be more correlated with changes in manual motor function. Furthermore, we hypothesized that baseline levels and longitudinal WM microstructural change in the tracts of interest would be most correlated with left hand and weakly or not correlated with right hand motor performance levels/change, since right-hand performance is well-trained and might be more adept at compensation.

## Materials and Methods

### Participants

Longitudinal motor and MRI data were taken from the Longitudinal Healthy Aging Brain (LHAB) database (Zöllig et al., 2011). We used data from the first four measurement occasions (baseline, 1-year follow-up, 2-year follow-up, 4-year follow-up). The baseline dataset included 232 participants (*M* age = 70.8; range: 64–87; females: 114). At each measurement occasion, participants completed an extensive battery of neuropsychological and psychometric cognitive and motor assessments and underwent brain imaging. The brain imaging session was conducted in close temporal proximity to the behavioral assessments [difference between behavioral and MRI assessments in days (M ± SD): baseline: 2.2 ± 5.2, 1-year follow-up: 2.6 ± 5.2, 2-year follow-up: 4.3 ± 13.0, 4-year follow-up: 4.6 ± 9.3]. Inclusion criteria for study participation at baseline were age ≥ 64, right-handedness, fluent German language proficiency, a score of ≥26 on the Mini Mental State Examination (MMSE; Folstein et al., 1975), no self-reported neurological disease of the central nervous system and no contraindications to MRI. The study was approved by the Ethics Committee of the Canton of Zurich. Participation was voluntary and all participants gave written informed consent in accordance with the declaration of Helsinki. Self-reported physical and mental health of the sample at baseline, as measured by the SF-12 (Ware et al., 1996), were 50.9 ± 7.4 (M ± SD) and 54.8 ± 6.3, respectively, which indicates above-average health compared to a normative population (Ware et al., 1995). As expected, sample means for these general health indicators slightly declined over time, but still indicated above-average health at 4-year follow-up (physical health score: 50.5 ± 6.9, mental health score: 53.1 ± 8.0, MMSE = 28.3 ± 1.3). At 4-year follow-up, the dataset still comprised 74.57% of the baseline sample (*n* = 173). As reported in other publications with this sample (Oschwald et al., 2019b; Malagurski et al., 2020), selectivity analyses showed that the participants remaining in the study at the 4-year follow-up did not substantially differ from the baseline sample in terms of age, education, physical and mental health, or head motion in the scanner.

For the present analysis, participants were excluded if either motor behavior or DW-MRI data were missing for all measurement occasions. With this criterion we were able to include 231 participants from the LHAB baseline sample (*M* age at baseline = 70.8; females: 113). Of those 231 participants, 172 were still participating at the 4-year follow-up. Participant characteristics at each measurement occasion are presented in Table 1.

**TABLE 1:** Participant characteristics of the full sample at baseline and at each follow-up wave.

**Variable**	**Baseline (*n* = 231)**			**1-year follow-up (*n* = 210)**			**2-year follow-up (*n* = 196)**			**4-year follow-up (*n* = 172)**		
	***n***	***M***	***SD***	***n***	***M***	***SD***	***n***	***M***	***SD***	***n***	***M***	***SD***
Baseline age (years)	231	70.82	5.08	210	70.92	5.15	196	70.64	4.80	172	70.12	4.43
Gender (m/f)	231	118/113	–	210	109/101	–	196	105/91	–	172	93/79	–
Education (1–3)	224	2.23	0.86	209	2.24	0.86	194	2.23	0.87	170	2.28	0.84
Mental health	211	54.78	6.26	194	54.60	6.40	183	54.54	6.26	158	54.68	5.74
Physical health	211	50.85	7.37	194	50.97	7.37	183	51.11	6.86	158	51.52	6.32
Head motion^*a*^	228	0.24	0.15	206	0.25	0.16	189	0.27	0.17	164	0.26	0.19

*m, male; f, female.*

*Education was measured on a scale from 1 to 3 (1 = high school with or without vocational education, 2 = higher education entrance qualification, business school or university of applied sciences, or 3 = university degree). Mental and physical health scores were computed based on the SF12 questionnaire, which participants filled out at home (Ware et al., 1996).*

*^*a*^Head motion was assessed at each measurement occasion. All other variables were assessed at baseline.*

### Brain Measures

#### MR Imaging

MRI measurements were conducted on a Philips Ingenia 3T scanner equipped with a commercial 32-element sensitivity encoding (SENSE) head coil array. The DW-MRI protocol employed an echo-planar (EPI) sequence [TR = 23.918 s, TE = 55 ms, FoV = 224 × 224 mm, acquisition matrix = 112 × 112, slice thickness = 2 mm, 75 contiguous slices, 2 mm^3^ isotropic voxel, flip angle = 90°, Echo Train Length (ETL) = 59, NSA = 1, SENSE factor *R* = 2.0]. One non-weighted image (*b*-value = 0 s/mm^2^) and 32 diffusion-weighted images (*b*-value of 1,000 s/mm^2^) were acquired. The diffusion-weighted directions were equally distributed in space. The same scanner and sequence were used at all measurement occasions.

#### MRI Data Preprocessing

To facilitate analysis, data were organized according to the brain imaging data structure (BIDS) (Gorgolewski et al., 2016). Diffusion data were processed with a nipype pipeline (v0.14.0) (Gorgolewski et al., 2011) using tools from MRtrix (3-rc2) (Tournier et al., 2012), FSL (v5.0.9) (Jenkinson et al., 2012), and ANTs (2.1.0) (Avants et al., 2011). The analysis code is publicly available: https://github.com/fliem/extract_FA, and a BIDS-Apps-compatible (Gorgolewski et al., 2017) software container to reproduce the analysis can be found here: http://hub.docker.com/r/fliem/extract_fa/.

The diffusion data were denoised (Veraart et al., 2016a,b) and corrected for eddy current distortions and head motion (Andersson and Sotiropoulos, 2016; Andersson et al., 2016). Subsequently, the data were bias-corrected (Tustison et al., 2010) and a white matter mask was created (Dhollander et al., 2016). Tensor maps were calculated (Veraart et al., 2013) and FA maps were derived (Basser et al., 1994; Westin et al., 1997). ANTs was used to register FA maps to the JHU-ICBM-FA template (included in FSL). Mean FA was extracted for tracts of the JHU WM tractography atlas (thresholded at 25% probability) for voxels with FA > 0.2 (Hua et al., 2008). The tracts considered here are: Forceps minor (FMIN), left and right hemispheric superior longitudinal fasciculi (SLF) and corticospinal tracts (CST). The size of the FMIN was 19407 voxels. For the SLF and CST, we averaged left and right hemispheres, weighted by the total number of voxels of the respective tract (SLF: 17657, CST: 10739). For the statistical analyses, we multiplied the FA values by 100 (now ranging from 0 to 100) to ensure a more intuitive interpretability of these scores for the reader and avoid any model estimation problems that might occur when bringing two domains together that are on very different scales.

#### Head Motion Control

As a means of ensuring sufficient quality of the data, we removed FA values for 56 individual observations (i.e., 7% of the total *N* of 787 observations) at which participants showed excessive head motion. As a measure of head motion, we used the summary statistic of average RMS motion as compared to the previous slice in a volume, which was calculated during preprocessing (Andersson and Sotiropoulos, 2016). Excessive values were defined as any value more than three median absolute deviations (MADs) above the median of the sample distribution across measurement occasions (Leys et al., 2013). We used the median as a reference, since it is more robust to the influence of extreme values than the mean.

### Motor Performance Measures

Motor performance was assessed by three motor tests described in detail below. Specifically, we used two tests to measure motor speed and one test to measure motor strength. First, all dependent values of interest were individually standardized by using the mean and standard deviation of the first measurement occasion, and then transformed into a T-statistic to achieve better interpretability.

#### Motor Speed

##### Grooved pegboard test

The grooved pegboard test (Merker and Podell, 2010) comprises a board with a cavity in the upper region, in which small metal sticks in key-like shapes (pegs) are stored. In the bottom area, the board has 25 holes (5 columns × 5 holes), each including a slit on the side to fit the key-shape of the pegs. However, these holes are oriented in a random fashion, such that the matching of a peg to the hole might require a turn of the peg. The test requires participants to sequentially place pegs into the corresponding holes in the board as quickly as possible. Participants are asked to complete the test with their left hand (proceeding from right to left) and with the right hand (proceeding from left to right). The dependent measure is the time the participant needs to place all pegs into the holes, separately measured for the left and the right hand.

##### Finger tapping

Finger tapping speed was assessed with the MLS (Motorische Leistungsserie; Schoppe, 1974). This test is based on a work panel that allows participants to perform a series of different uni- or bimanual tasks designed to assess fine motor skills. Specifically, the finger tapping task required participants to tap as quickly as possible on one of two small squares located on the bottom left and right of the panel with the tip of a pen. Performance was separately measured for the right and the left hand and participants were asked to target the square that was located on the same side as their respective hand. The dependent measure of interest was the number of taps within 32 s, separately measured for the left and the right hand.

#### Motor Strength

##### Grip force

Grip force was measured with a hydraulic hand dynamometer (Merker and Podell, 2010) (Model SH5001, Sae-han Corporation, Korea) to assess the isometric maximum grip force of the right and the left hand. Participants were instructed to sit upright, with their feet positioned flat on the ground, their shoulders and forearm in a neutral position, their elbow in 90° flexion and the wrist in an extension of 0° to 30°. Participants were asked to press the dynamometer for 4 s with maximum force, beginning with the dominant hand. After a 30 s break, they had to switch hands and repeat the task with the non-dominant hand. Overall, three repetitions were conducted for each hand. If the maximum grip force in one hand was higher in the third as compared to the first two rounds, data collection was continued until the final measurement was smaller than the previous one. The dependent measure of interest was the average grip force across the three highest measurements, separately computed for the left and the right hand.

### Covariates

To control for potential confounding influences, we included age at baseline (Age_*base*_), level of education (on a scale from 1 to 3; 1 = high school with or without vocational education, 2 = higher education entrance qualification, business school or university of applied sciences, or 3 = university degree) and gender (0 = female, 1 = male) as covariates on the intercept and slope terms in all statistical analyses. Furthermore, for the FA models, we also included head motion in the scanner as a time-varying covariate on the manifest indicators at each measurement occasion. To facilitate model interpretation, age was centered at 70 years (median of the sample), and education at level 2. Head motion was left uncentered, since a value of zero was meaningful (i.e., reflecting no head motion).

### Statistical Analysis

All statistical analyses were run in R version 3.3.3 (R Core Team, 2019). Outlier correction in each motor performance measure was done using a cut-off of three MADs above or below the median of the sample distribution across measurement occasions, resulting in the removal of 58 individual values [i.e., between 0.1% and 3.2-% of the total *N* of observations for each test; Grooved Pegboard left: *n* (%) = 25 (3.2%) and right *n* (%) = 21 (2.7%); Finger tapping left: *n* (%) = 3 (0.4%) and right *n* (%) = 7 (0.9%); Grip Force left: *n* (%) = 1 (0.1%) and right *n* (%) = 1 (0.1%)]. We refrained from outlier control in FA measures, since FA can largely vary between individuals (e.g., Veenith et al., 2013), and no clear consensus exists on normative cut-offs. However, we excluded individual observations with excessive head motion values (see section on “Head motion control” above).

#### Latent Growth Curve Modeling (LGC)

In the present study, we first used univariate LGC to model change in FA and motor performance measures individually, and bivariate LGC to model cross-domain interactions between these measures. We estimated the LGC models in the SEM framework using the *lavaan* package version 0.5-23.1097 (Rosseel, 2012) in R.

##### Univariate models

**FIGURE 1:**
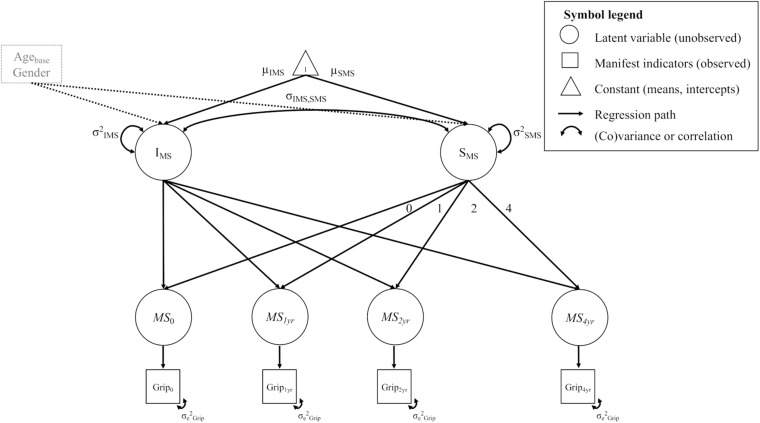
Example diagram of a univariate LGC model for motor strength (MS) in the grip force test (Grip). For a detailed description see methods section (univariate models). All unlabeled paths are fixed to 1. Parameters with the same label are fixed to be equal. The manifest indicator intercepts are not shown for visual clarity. Intercept and slope variance is controlled for age at baseline (Age_*base*_) and gender.

We estimated separate univariate LGC models for FA in each of the WM tracts and for motor function in the tests assessed. For each univariate LGC we estimated the (1) initial level, i.e., the intercept of the measure of interest, (2) its rate of (linear) change, i.e., the slope, and (3) the association between the initial level and the rate of change of the measure of interest. To avoid confounding of initial level/rate of change estimation, we added baseline age and gender as covariates to all models. For the univariate FA models, we additionally added head motion as a time-varying covariate. To illustrate, Figure 1 shows a path diagram of a univariate LGC model for motor strength.

From bottom to top, the Figure shows the observed measurements of grip force at each time point (*Grip*_0_… *Grip*_4__*yr*_). These measurements load on the latent estimates of motor strength (*Ms*_0_…*Ms*_4__*yr*_). In other words, the motor strength variables represent latent estimations of grip force at each measurement occasion, separated from measurement error. Further, a latent intercept (*I*) and slope factor (*S*) was estimated on top of the latent estimates of motor strength, to capture initial levels and overall rate of change across time. The means of these factors reflect the average baseline value (μ*_*I*_*) and change (μ*_*S*_*) in a variable across the entire sample (i.e., fixed effects). The variances of these latent factors reflect the variability between persons (i.e., random effects) in their individual baseline values (*σ^2^_*I*_*) and change trajectories (*σ^2^_*S*_*). We estimated the loadings of the change slope to reflect a linear change trajectory (i.e., slope loadings of 0, 1, 2, 4). As is the standard in longitudinal SEM modeling, we treated missing values as missing at random (MAR; Little, 1995) and retained them in the model by using the full information maximum likelihood estimation (FIML; Finkbeiner, 1979; Schafer and Graham, 2002) to deal with incomplete data.

##### Bivariate models

**FIGURE 2:**
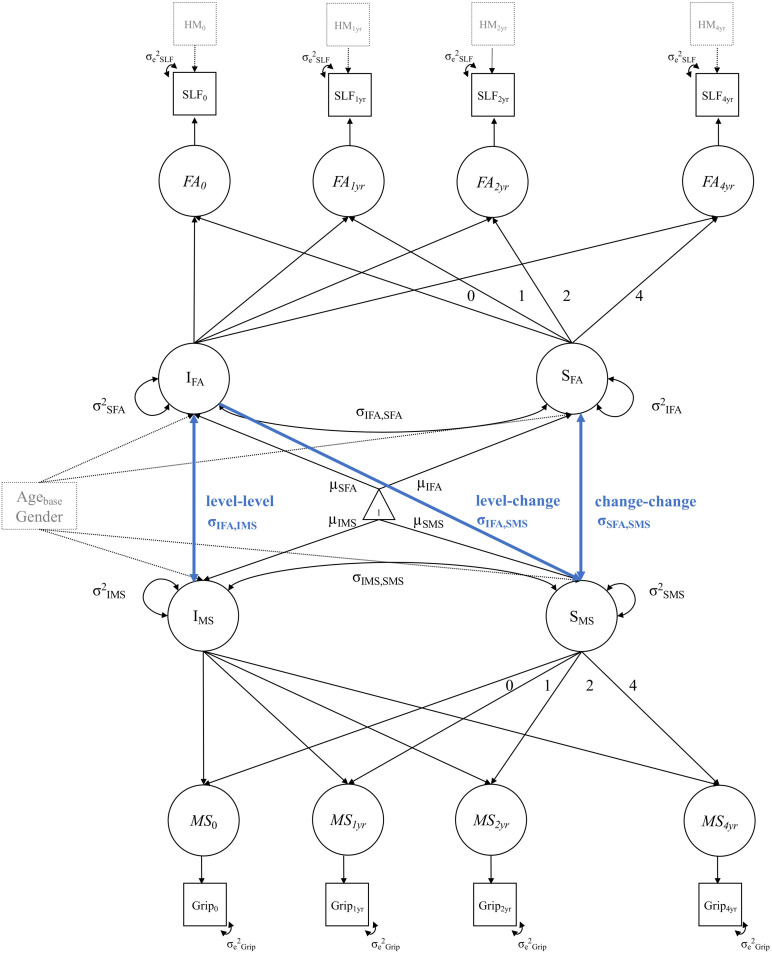
Example diagram of a bivariate LGC model for motor strength (MS) in the grip force test (Grip) and WM FA in the SLF. Blue paths illustrate the cross-domain associations between (1) initial WM FA and motor performance at study baseline (level–level), (2) initial WM FA and subsequent change in motor performance (level-change), change in WM FA and change in motor performance (change–change). Single-headed arrows represent regression effects and double-headed arrows represent (co)variances and correlations. Circles represent latent, unobserved variables and squares represent manifest, observed variables. Triangles stand for constants, such as means and intercepts. All unlabeled paths are fixed to 1. Parameters with the same label are fixed to be equal. The manifest indicator intercepts are not shown for visual clarity. Intercept and slope variance for motor strength and WM FA is controlled for age at baseline (Age_*base*_) and gender. The observed variables of WM FA in the SLF are controlled for head motion (HM) at each measurement occasion.

We estimated a series of bivariate LGC models, combining the univariate LGC models for each of the WM tracts and for each of the motor function tests, resulting in 18 models. To illustrate, Figure 2 shows a path diagram of a bivariate LGC model for motor strength (i.e., grip force test) and WM FA in the SLF.

In each bivariate LGC model, we estimated the following cross-domain interactions between FA and motor function (blue pathways in Figure 2): (1) the level-level (i.e., intercept-intercept) correlation, to investigate the association between baseline FA values in each of the WM tracts and baseline motor function in each of the motor function tests. As an example, a positive level-level association would suggest that at study initiation, individuals with higher WM FA show better motor performance than individuals with lower WM FA values. (2) The level-change (i.e., intercept-slope) correlation to examine the association between baseline FA values and motor performance changes. As an example, a positive level-change association would indicate that people with higher WM FA at baseline show reduced motor performance declines over the study period (3) the change-change (i.e., slope-slope) correlation, to investigate the association between change in WM FA and change in motor function. As an example, a positive change-change association would mean that people with steeper declines in WM FA also show steeper declines in motor performance over the study period. Note that these examples are formulated based on the assumption that individuals experience declines in WM FA and motor performance over time.

We only evaluated these longitudinal cross-domain effects, if there was sufficient variance in the intercepts and slopes of the respective univariate models of the combined variables. We defined sufficient variance as intercept or slope variance that is significantly (*p* <.05) different from zero, suggesting that individuals show substantial heterogeneity with regards to their baseline levels or their longitudinal change trajectories in WM FA or motor performance. While intercept variance was substantial in all univariate models, this was not the case for slope variance in some of the models. As a significance test relies on an arbitrary cut-off, we additionally calculated effective curve reliability (ECR) for each univariate model. ECR is a reliability index for LGC model slope variance that can be interpreted as a standardized effect size statistic of the slope variance (Kelley and Preacher, 2012; Brandmaier et al., 2018). It is computed as the slope variance scaled as a proportion of the sum of slope variance and slope measurement error. ECR ranges from 0 to 1, with larger values reflecting increased true population slope variance and/or increased study design precision (and thus reduced effective error) (Brandmaier et al., 2018).

##### Evaluation of model fit

Overall model fit was evaluated by the χ^2^ test, specifically, by the ratio of the χ^2^ test statistic to the respective degrees of freedom (Jöreskog and Sörbom, 1993). Furthermore, the Comparative Fit Index (CFI; Bentler, 1990), and the root mean square error of approximation (RMSEA; Steiger and Lind, 1980) were used to evaluate goodness-of-fit. Good model fit was defined as a ratio of χ^2^/*df* ≤ 2, CFI > 0.97, RMSEA ≤ 0.05, and adequate fit was defined as χ^2^/*df* ≤ 3, CFI > 0.95, RMSEA between 0.05 and 0.08 (see Jöreskog and Sörbom, 1993; Schermelleh-Engel et al., 2003). Models were compared using the difference χ^2^ test (for nested models) and the sample size adjusted Bayesian Information Criterion (BIC; Raftery, 1995). The BIC is not interpretable in isolation, however, in model comparisons, smaller values indicate a closer fit of the model to the data (Kass and Raftery, 1995; Raftery, 1995). Given that we tested a large number of hypotheses, we applied a correction for multiple comparisons using the False Discovery Rate (FDR) correction, to reduce the likelihood of false positive findings, i.e., Type 1 errors (Benjamini and Hochberg, 1995). The FDR correction was applied to each LGC model separately, and across all effects of interest within the structural part of the model. For the univariate LGC models, we included the means and variances of intercept and slope, covariate effects and within-domain correlations of intercept and slope as effects of interest. For the bivariate LGC models, we only considered cross-domain correlations between intercept and slopes as effects of interest.

## Results

Raw FA declined annually for the FMIN and SLF by –0.20 ± 1.18% and –0.56 ± 0.88%, respectively. In the CST, annual increases were observed in average FA values (0.25 ± 0.92%). This effect, however, did not manifest itself in the univariate LGC models described below. Potential explanations for this divergence between raw change and modeled change values will be addressed in the discussion. Average annual declines were observed in all motor function tests [annual percentage change (APC) ranging between –0.24% and 1.21%]. Grip force and tapping speed of the left hand declined more strongly compared to the right (dominant) hand, while we observed the reversed pattern (higher annual decline in the right hand) for performance in the pegboard test. Table 2 contains a detailed overview of the raw annual change and APC in FA and motor functioning.

**TABLE 2:** Raw annual change and annual percentage change of WM microstructure and motor function.

**Variable/change score**	**Raw annual change**			**APC (%)**	
	***n***	***M***	***SD***	***M***	***SD***
**WM microstructure (raw FA scores × 10^2^)**					
FMIN	197	–0.07	0.42	–0.20	1.18
SLF	197	–0.20	0.31	–0.56	0.88
CST	197	0.13	0.50	0.25	0.92
**Motor function (standardized *T*-scores)**					
Pegboard (left)	202	–0.22	2.79	–0.24	7.16
Pegboard (right)	206	–0.46	2.96	–0.78	7.38
Tapping (left)	205	–0.45	2.37	–1.09	5.07
Tapping (right)	204	–0.32	2.53	–0.67	6.02
Grip force (left)	210	–0.63	1.63	–1.21	3.68
Grip force (right)	210	–0.49	1.74	–0.91	4.22

*Raw annual change, raw annual change score, calculated as [(Value at last measurement occasion in the study-Value at baseline)/total years in study]; APC, annual percentage change, calculated as [(Value at last measurement occasion in the study/Value at baseline)^1/(total years in study)^-1]^∗^100.*

### Univariate LGC Models: FA

We fit univariate LGC models for mean FA in each of three WM tracts (FMIN, SLF, CST). Model fit statistics and parameter estimates are shown in Table 3.

**TABLE 3:** Parameter estimates and model fit statistics of best fitting univariate LGC models for WM FA.

		**Mean (μ)**			**Variance (σ*^2^*)**					**Model fit**			
**LGC model/WM tract**		**Estimate**	***SE***	***p* value**	**Estimate**	***SE***	***p* value**	**ECR**	**χ^2^(*df*)**	**χ^2^/*df***	**RMSEA [95% *CI*]**	**CFI**	**BIC**
FMIN	Intercept	37.36	0.24	**<0.001**	4.30	0.44	**<0.001**		42.695 (24)	1.78	0.058 [0.028–0.086]	0.983	3257.441
	Slope	–0.15	0.06	**0.014**	0.04	0.01	**0.001**	0.44					
SLF	Intercept	36.19	0.21	**<0.001**	3.27	0.33	**<0.001**		67.697 (24)	2.82	0.089 [0.064–0.114]	0.960	3023.458
	Slope	–0.2	0.05	**<0.001**	0.02	0.01	**0.024**	0.30					
CST	Intercept	54.52	0.27	**<0.001**	3.90	0.44	**<0.001**		31.733 (24)	1.32	0.037 [0.000–0.069]	0.988	3634.188
	Slope	–0.04	0.08	0.628	0.00^*a*^	0.00	**<0.001**	– ^*b*^					

*ECR, Effective Curve Reliability; RMSEA, Root Mean Square Error of Approximation; CFI, Comparative Fit Index; BIC, Bayesian Information Criterium. Parameter estimates are unstandardized, and adjusted for effects of age at baseline, and gender (on intercept and slope), and for head motion at each measurement occasion. FA values are raw scores, multiplied by 100. Results that were significant after FDR correction for multiple comparisons (*p* < 0.05) are highlighted in bold font.*

*^*a*^The slope variance was constrained to be positive, which resulted in a slope variance of zero.*

*^*b*^ECR could not be calculated because the slope variance was zero.*

Fit statistics ranged from adequate to good for all models [χ^2^_(_*_24_*_)_ = 31.733–67.697, χ^2^/*df* = 1.32–2.82, RMSEA = 0.037–0.089, CFI = 0.960–0.988]. With regards to the parameter estimates, substantial individual differences (i.e., significant intercept variance) were observed for baseline mean FA in all WM tracts. On average, mean scaled FA declined in the FMIN and SLF (–0.15 and –0.20 per year). In contrast, as we hypothesized, no substantial average FA changes were observed in the CST. Moreover, substantial interindividual differences in longitudinal change (i.e., significant slope variance) were observed for the FMIN and SLF. However, the univariate LGC model for the CST initially converged with a negative slope variance. Constraining the CST slope variance to a positive value resulted in the estimation of a slope variance of zero (see Table 3), suggesting the absence of interindividual differences in longitudinal average FA change trajectories for the CST. Accordingly, ECR could not be calculated for the CST. As sufficient variance in change trajectories is necessary to estimate cross-domain parallel change correlations, only the FMIN and SLF were retained for the bivariate modeling of change-change associations. ECR for the FMIN and SLF were 0.44 and 0.30 (see Table 3), respectively, suggesting a small to medium effect size of the slope variance for these tracts (Cohen, 1988, 1992). Covariance between intercept and slope was not significant for any of the WM tracts.

With regard to covariate effects on intercept and slope (see Table 4), baseline age was significantly associated with baseline mean FA in the FMIN and SLF, in the direction that older individuals had lower FA values in these tracts at baseline than their younger peers. Baseline age was also significantly related to changes in mean FA over time in the FMIN and CST, suggesting that participants showed accelerated annual FA decline with increasing age (mean scaled FA of –0.01 and –0.02). Specifically, as no significant main effect of mean FA change was observed for the CST, this result suggests that FA decline was predominantly observed for the older participants in the sample. Gender had a significant effect on average baseline FA in the FMIN, and FA changes in the CST: Male participants had lower mean FA in the FMIN at baseline (–0.64, *SE* = 0.29, *p* = 0.026), and showed less annual mean FA change in the CST (0.22, *SE* = 0.06, *p* < 0.001) than female participants.

**TABLE 4:** Effects of Age at baseline and Gender on intercept and slope of best fitting univariate LGC models for WM FA.

		**Age_*base*_**			**Gender**		
**Tract**		**Estimate**	***SE***	***p*-value**	**Estimate**	***SE***	***p*-value**
FMIN	Intercept	–0.18	0.03	**<0.001**	–0.64	0.29	**0.026**
	Slope	–0.01	0.01	**0.029**	0.05	0.05	0.316
SLF	Intercept	–0.13	0.03	**<0.001**	0.36	0.25	0.148
	Slope	–0.01	0.00	0.122	0.01	0.04	0.692
CST	Intercept	0.00	0.03	0.892	0.51	0.29	0.075
	Slope	–0.02	0.01	**0.017**	0.22	0.06	**<0.001**

*Age_*base*_, age at baseline. Parameter estimates are unstandardized. Results that were significant after FDR correction for multiple comparisons (*p* < 0.05) are highlighted in bold font.*

Finally, we also investigated the impact of head motion at each measurement occasion (see Table 5). Head motion was significantly associated with FA in all WM tracts on at least one measurement occasion, such that more motion in the scanner was associated with significantly lower average FA. This effect was most consistently observed for the CST. Note that one unit increase in head motion amounts to almost five times the average head motion present in the sample (from 0.24 to 0.27 across measurement occasions; cf. Table 1).

**TABLE 5:** Effects of head motion on FA at each measurement occasion of best fitting univariate LGC models for WM FA.

	**Head Motion_*base*_**			**Head Motion_1year_**			**Head Motion_2years_**			**Head Motion_4years_**		
**Tract**	**Estimate**	***SE***	***p*-value**	**Estimate**	***SE***	***p*-value**	**Estimate**	***SE***	***p*-value**	**Estimate**	***SE***	***p*-value**
FMIN	–1.76	0.57	**0.002**	–0.49	0.46	0.289	–0.70	0.46	0.128	–1.10	0.73	0.134
SLF	–0.90	0.49	0.069	–2.02	0.40	**<0.001**	–1.73	0.39	**<0.001**	–0.52	0.60	0.384
CST	–2.51	0.83	**0.002**	–2.11	0.68	**0.002**	–2.29	0.66	**0.001**	–0.89	0.95	0.346

*Base, baseline. Parameter estimates are unstandardized. Results that were significant after FDR correction (*p* < 0.05) are highlighted in bold font.*

### Univariate LGC Models: Motor Function

We fit univariate LGC models to estimate longitudinal performance change in each of the motor function tests (motor speed: pegboard, tapping; motor strength: grip force), and separately for each hand. Model fit statistics and parameter estimates of these models are presented in Table 6.

**TABLE 6:** Parameter estimates and model fit statistics of best fitting univariate LGC models for motor function.

			**Mean (*μ*)**			**Variance (*σ ^2^*)**				**Model fit**				
**LGC model/WM tract**			**Estimate**	***SE***	***p*-value**	**Estimate**	***SE***	***p*-value**	**ECR**	**χ ^2^(*df*)**	**χ ^2^/*df***	**RMSEA [95% *CI*]**	**CFI**	**BIC**
Motor Speed														
	Pegboard (*l*)	Intercept	51.06	0.84	**<0.001**	60.50	7.48	**<0.001**		15.972 (12)	1.33	0.038 [0.000–0.082]	0.993	6907.057
		Slope	–0.31	0.21	0.138	0.27	0.44	0.545	0.09					
	Pegboard (*r*)	Intercept	51.83	0.84	**<0.001**	59.07	7.44	**<0.001**		19.685 (12)	1.64	0.053 [0.000–0.093]	0.987	6972.606
		Slope	–0.56	0.21	**0.007**	0.25	0.46	0.584	0.09					
	Tapping (*l*)^*a*^	Intercept	47.60	0.95	**<0.001**	85.14	8.81	**<0.001**		27.957 (13)	2.15	0.071 [0.034–0.107]	0.980	6875.361
		Slope	–0.53	0.18	**0.003**	0.06	0.29	0.842	0.03					
	Tapping (*r*)	Intercept	47.15	0.86	**<0.001**	68.98	7.68	**<0.001**		23.735 (12)	1.98	0.065 [0.024–0.103]	0.984	6813.738
		Slope	–0.44	0.18	**0.014**	0.35	0.33	0.289	0.15					
Motor Strength														
	Grip Force (l)	Intercept	42.31	0.56	**<0.001**	30.21	3.27	**<0.001**		31.070 (12)	2.59	0.083 [0.048–0.119]	0.986	6164.275
		Slope	–0.45	0.13	**<0.001**	0.50	0.16	**0.002**	0.47					
	Grip Force (r)	Intercept	42.20	0.56	**<0.001**	28.82	3.19	**<0.001**		21.785 (12)	1.82	0.059 [0.013–0.099]	0.993	6260.735
		Slope	–0.39	0.14	**0.005**	0.80	0.20	**<0.001**	0.60					

*ECR, Effective Curve Reliability; RMSEA, Root Mean Square Error of Approximation; CFI, Comparative Fit Index; BIC, Bayesian Information Criterium; l, left; r, right. Parameter estimates are unstandardized, and adjusted for effects of age at baseline, and gender (on intercept and slope). Motor function values are T-distributed for all tests (pegboard, tapping and grip force), with higher values reflecting better performance. Results that were significant after FDR correction for multiple comparisons (*p* < 0.05) are highlighted in bold font.*

*^*a*^The slope variance of this model was constrained to be positive and the intercept-slope covariance was fixed to zero.*

Fit statistics ranged from adequate to good for all models [χ^2^_(__12__)_ = 15.972–31.070, χ^2^/df = 1.33–2.59, RMSEA = 0.038–0.083, CFI = 0.980–0.993]. Substantial interindividual differences were observed for motor performance at baseline in all tests. On average, motor performance declined in all tests (between –0.39 and –0.56 per year) but one (Pegboard left hand performance). Mirroring the raw performance changes reported in Table 2, larger declines were observed for left hand than right hand motor function. Regarding longitudinal change trajectories, substantial interindividual variance was only observed for motor strength in the grip force test (left and right hand). In contrast, the slope variance was not significantly different from zero for any of the motor speed tests^1^. Mirroring this result from the significance test, ECR and therefore slope variance effect size was below the cut-off of a small effect size for the motor speed tests (between 0.03–0.15) and of medium size for the Grip Force test (left hand: 0.47, right hand: 0.60) (Cohen, 1988, 1992). Due to the lack of substantial change variability in the motor speed tests, only the grip force test (left and right hand) will be retained for the estimation of change-change associations in the bivariate modeling section. The covariance between intercept and slope was significant only in the grip force test (left hand: –1.15, *SE* = 0.53, *p* = 0.029, right hand: –1.57, *SE* = 0.60, *p* = 0.009), in the direction that individuals with higher motor function at baseline tended to show accelerated declines in motor function over 4 years.

Baseline age was significantly negatively associated with motor function across all tests, such that older individuals had a lower performance at baseline than younger peers (Table 7). In addition, baseline age was significantly negatively associated with performance changes in two motor speed tests (pegboard right hand: –0.13 and left hand tapping: –0.07), indicating that older participants’ motor function declined more rapidly as compared to their younger peers.

**TABLE 7:** Effects of Age at baseline and Gender on intercept and slope of best fitting univariate LGC models for motor function.

			**Age_*base*_**			**Gender**		
**Tract**			**Estimate**	***SE***	***p*-value**	**Estimate**	***SE***	***p*-value**
Motor Speed								
	Pegboard (*l*)	Intercept	–0.86	0.12	**<0.001**	–0.57	1.17	0.629
		Slope	–0.03	0.03	0.374	–0.27	0.28	0.345
	Pegboard (*r*)	Intercept	–0.86	0.12	**<0.001**	–2.26	1.17	0.053
		Slope	–0.13	0.03	**<0.001**	–0.25	0.28	0.370
	Tapping (*l*)	Intercept	–0.43	0.13	**0.001**	5.43	1.32	**<0.001**
		Slope	–0.07	0.03	**0.006**	0.21	0.24	0.387
	Tapping (*r*)	Intercept	–0.60	0.12	**<0.001**	6.16	1.20	**<0.001**
		Slope	–0.01	0.03	0.740	0.17	0.25	0.490
Motor Strength								
	Grip Force (*l*)	Intercept	–0.47	0.08	**<0.001**	15.34	0.78	**<0.001**
		Slope	–0.01	0.02	0.632	–0.34	0.17	0.045^*a*^
	Grip Force (*r*)	Intercept	–0.45	0.08	**<0.001**	15.66	0.77	**<0.001**
		Slope	–0.01	0.02	0.549	–0.22	0.19	0.246

*Age_*base*_, age at baseline; l, left; r, right. Parameter estimates are unstandardized. Results that were significant after FDR correction for multiple comparisons (*p* < 0.05) are highlighted in bold font.*

*^*a*^No longer significant (*p* < 0.05) after correction for multiple comparisons.*

Gender was significantly related with motor function at baseline, such that male participants showed better motor performance in the tapping (left hand: 5.43, *SE* = 1.32, *p* < 0.001; right hand: 6.16, *SE* = 1.20, *p* < 0.001) and grip force tests (left hand: 15.34, *SE* = 0.78, *p* < 0.001; right hand: 15.66, *SE* = 0.77, *p* < 0.001). In addition, male participants showed steeper declines in left hand grip force performance over 4 years than female participants (–0.34, *SE* = 0.17, *p* = 0.045), however, this effect was no longer significant after correction for multiple comparisons.

### Bivariate LGC Models: FA and Motor Function

We fit bivariate LGC models to estimate cross-domain relationships between mean WM FA in each of the three WM tracts and motor function in each of the six motor tests, resulting in overall 18 separate models. Fit statistics of these models are presented in Table 8, and standardized parameter estimates are shown in Table 9.

**TABLE 8:** Model fit statistics of bivariate LGC models (for parameter estimates see Table 9).

**WM tract**	**Motor function**	**χ^2^ (*df*)**	**χ^2^/*df***	**RMSEA [95% *CI*]**	**CFI**	**BIC**
FMIN	Pegboard (*l*)	94.089 (67)	1.40	0.042 [0.019–0.061]	0.984	8421.788
	Pegboard (*r*)	79.877 (67)	1.19	0.029 [0.000–0.050]	0.992	8488.734
	Tapping (*l*)	110.001 (68)	1.62	0.052 [0.033–0.069]	0.977	8387.129
	Tapping (*r*)	92.599 (67)	1.38	0.041 [0.017–0.060]	0.986	8329.705
	Grip Force (*l*)	102.529 (65)	1.58	0.050 [0.030–0.068]	0.985	7684.128
	Grip Force (*r*)	96.723 (65)	1.49	0.046 [0.025–0.064]	0.987	7779.699
SLF	Pegboard (*l*)	113.834 (67)	1.70	0.055 [0.037–0.072]	0.972	8188.823
	Pegboard (*r*)	107.235 (67)	1.60	0.051 [0.032–0.068]	0.976	8255.944
	Tapping (*l*)	122.849 (68)	1.81	0.059 [0.042–0.076]	0.970	8156.749
	Tapping (*r*)	109.217 (67)	1.63	0.052 [0.034–0.070]	0.977	8098.732
	Grip Force (*l*)	123.705 (65)	1.90	0.063 [0.046–0.079]	0.976	7453.513
	Grip Force (*r*)	131.512 (65)	2.02	0.067 [0.050–0.083]	0.973	7549.944
CST	Pegboard (*l*)	79.414 (67)	1.19	0.028 [0.000–0.050]	0.990	8802.741
	Pegboard (*r*)	71.231 (67)	1.06	0.017 [0.000–0.043]	0.996	8868.370
	Tapping (*l*)	86.920 (68)	1.28	0.035 [0.000–0.055]	0.986	8770.575
	Tapping (*r*)	79.729 (67)	1.19	0.029 [0.000–0.050]	0.991	8709.157
	Grip Force (*l*)	96.105 (66)	1.46	0.044 [0.023–0.063]	0.985	8061.218
	Grip Force (*r*)	99.550 (66)	1.51	0.047 [0.026–0.065]	0.983	8154.132

*RMSEA, Root Mean Square Error of Approximation; CFI, Comparative Fit Index; BIC, Bayesian Information Criterium; l, left; r, right.*

**TABLE 9:** Results of bivariate LGC models (level-level, level-change, change-change).

**Correlation**	**WM tract**	**Motor speed**				**Motor strength**	
		**Pegboard (*l*)**	**Pegboard (*r*)**	**Tapping (*l*)**	**Tapping (*r*)**	**Grip Force (*l*)**	**Grip Force (*r*)**
Level-level	FMIN	0.16 (0.08) *	0.13 (0.07)	0.19 (0.07)**	0.13 (0.07)	0.13 (0.07)	0.16 (0.07)^*a^
	SLF	0.14 (0.08)	0.10 (0.08)	0.14 (0.07)	0.02 (0.08)	0.01 (0.07)	0.04 (0.08)
	CST	–0.03 (0.08)	–0.01 (0.07)	–0.05 (0.07)	–0.04 (0.07)	–0.07 (0.07)	–0.14 (0.07)
Level-change	FMIN	–	–	–	–	0.00 (0.13)	–0.12 (0.11)
	SLF	–	–	–	–	0.07 (0.13)	–0.05 (0.12)
	CST	–	–	–	–	–0.00 (0.12)	–0.03 (0.11)
Change-change	FMIN	–	–	–	–	–0.13 (0.19)	–0.06 (0.16)
	SLF	–	–	–	–	0.01 (0.23)	0.04 (0.20)
	CST	–	–	–	–	–	–

Parameter estimates are standardized, with standard errors in parentheses. Results that were significant (*p < 0.05, **p < 0.01) are marked with stars. Results that were still significant after FDR correction for multiple comparisons are highlighted in bold font.

*FMIN, Forceps Minor; SLF, Superior Longitudinal Fasciculus; CST, Corticospinal Tract; l, left; r, right.*

*^*a*^No longer significant (*p* < 0.05) after correction for multiple comparisons.*

Model fit statistics ranged from adequate to good for all models [χ^2^_(_*_65__–__68_*_)_ = 71.231 - 131.512, χ^2^/*df* = 1.06–2.02, RMSEA = 0.017–0.067, CFI = 0.970–0.996]. First, in each of the 18 bivariate LGC models, we estimated level–level (i.e., intercept–intercept) correlations between baseline mean FA values and baseline motor function. The results revealed a significant positive correlation for the FMIN and motor speed performance: higher baseline mean FA in the FMIN was associated with faster motor performance in the pegboard (fully standardized estimate: 0.16, *SE* = 0.08, *p* = 0.035) and tapping tests (fully standardized estimate: 0.19, *SE* = 0.07, *p* = 0.006) for the left, but not for the right hand. These estimates correspond to a small to typical effect size if compared to the norms in interindividual difference research (Gignac and Szodorai, 2016). We also observed a positive correlation for the FMIN and right-hand grip force performance, however, this result was no longer significant after correction for multiple comparisons. None of the other level–level correlations were significant.

In addition, we estimated level-change (i.e., intercept-slope) correlations to investigate the association between baseline mean FA values and motor performance changes. As the motor speed tests did not show sufficient slope variance, we only estimated level-change correlations for the motor strength tests (grip force left and right hand). None of these results were significant.

Finally, to investigate the association between changes in mean WM FA and changes in motor function, we estimated change–change (i.e., slope–slope) correlations between motor strength in the grip force test and mean FA in those WM tracts that showed sufficient slope variance (FMIN, SLF). Again, none of these correlations were significant.

## Discussion

In the current study we followed > 200 healthy older adult participants over 4 years of longitudinal DW-MRI and manual motor performance assessments. To optimally harvest insights from longitudinal data, we conducted our analyses using LGC models in the SEM framework, which enable the separation of interindividual variability from intraindividual change trajectories. We specifically targeted the FMIN, SLF, and CST for their purported roles in motor and cognitive function. Cross sectional associations with age at baseline indicate that mean FA values in these tracts as well as motor performance indicators are lower in older individuals, similar to what has been shown previously (Ruff and Parker, 1993; Jiménez-Jiménez et al., 2011; Bennett and Madden, 2014; Cox et al., 2016). We observed longitudinal declines over time in all measures except for average FA in the CST. Baseline age was also related to changes in mean FA over time in the FMIN and CST, suggesting that changes accelerated with advancing age in the FMIN. In case of the CST, this finding suggests that longitudinal decline is restricted to the oldest participants, as no decline was observed in average FA for the overall sample in this tract. We found that FMIN tract average FA values correlate positively with motor speed and motor strength measures, suggesting that maintenance of WM structure in the anterior corpus callosum is associated with better manual motor function. We did not observe significant longitudinal level-change or change-change associations, suggesting that – at least over the timescale studied here – age-related baseline levels and changes in WM microstructure within these tracts do not associate with changes in motor speed and grip force.

### Longitudinal Change in and Cross-Sectional Age Effects on Motor Speed and Motor Strength

Our LGC models for motor function reveal both longitudinal declines over time and cross-sectional effects of age, consistent with previous literature (Kallman et al., 1990; Ruff and Parker, 1993; Vianna et al., 2007). In addition, our results point to an acceleration of motor performance declines in older individuals. In light of the recent proposition that changes in grip strength are not simply an index of muscle mass loss but should be viewed as a marker of brain health given the complex neural circuitry that it engages (Carson, 2018), the loss of left-hand grip strength with an annual decline rate of 1.2% (Table 2) is particularly interesting. While some studies have reported greater grip strength loss with age in the right hand (Teixeira, 2008), others have shown more selective loss of neural control of the left (non-dominant) hand (Sale and Semmler, 2005). Here, we observed greater declines for the left hand, supporting our hypothesis of right-hand performance preservation due to stronger lifelong practice of the dominant hand. With respect to gender, we expectedly found significant cross-sectional effects for baseline grip force and tapping performance, with males having higher grip strength and faster tapping speeds. This is in accordance with previous work (Hubel et al., 2013; Yorke et al., 2015; Wong, 2016).

### Longitudinal Change in and Cross-Sectional Age Effects on Fractional Anisotropy

We found that both FMIN and SLF WM indices significantly declined over time, whereas CST did not. However, age was significantly negatively associated with CST change, implying that CST changes over time only occurred for the oldest participants in our sample. It should be noted that, in the case of the CST, the results from the univariate LGC model diverged from the annual change computed based on the raw FA values. While the modeled change values suggested stability in mean FA over 4 years, annual mean FA increases were observed in the raw values (0.25% ± 0.92%). This divergence most likely is a result of the inclusion of head motion and baseline age as covariates in the LGC models, but not in the calculation of the raw change values. While head motion had a significant impact on all WM tracts, the CST was most affected. Especially, head motion had an impact on the CST at the first three measurement occasions (i.e., by underestimating mean FA values), but not at the last occasion, which was also spaced further apart and thus had more weight for the change calculation. If not adjusted for, this imbalance in the FA estimation might result in the finding of FA increases over time instead of stability. The strict control for head motion, both by excluding observations with extreme motion, and including head motion as a covariate into the estimation of FA, is a major strength of this study. It has been previously reported that participant’s head movements in the scanner can result in biased FA estimates (Yendiki et al., 2014; Baum et al., 2018). Despite this common knowledge, head motion is often neglected in DW-MRI studies.

We have recently shown that a global WM decline factor is not a good fit to our data (Oschwald et al., 2019b). Lövdén et al. (2013) have also reported that aging differentially impacts individual WM tracts. Further, studies investigating gray and WM volume and WM diffusion changes with age largely provide evidence for the “last in, first out” hypothesis. This conceptual model proposes that tracts which are later to mature developmentally will be more sensitive to aging effects (Raz, 2000, 2001; Bender et al., 2016). Some studies have shown that primary motor and somatosensory cortex volumes are relatively spared by aging (Raz et al., 1997). In contrast, other studies have suggested disproportionate age effects on pre- and postcentral gyrus volume (Good et al., 2001; Taubert et al., 2020), cortical thickness (Salat et al., 2004), iron and myelin content (Taubert et al., 2020). It should be noted, however, that these studies were all cross-sectional and did not quantify longitudinal changes. In the current longitudinal investigation, we see more robust declines in WM microstructure for the FMIN and SLF than for the CST, which is consistent with the idea of a higher susceptibility of association and commissural fibers for detrimental effects of aging (Madden et al., 2012; Bender et al., 2016).

### Cross-Sectional and Longitudinal Brain-Behavior Associations

With regard to cross-sectional brain-behavior associations at baseline, left hand pegboard and tapping speed measures were significantly correlated with FMIN FA, with higher average FA values being associated with faster performance. There was also a trend for a level-level association between mean FA in the FMIN and right-hand grip force, but this association did not survive correction for multiple comparisons. We have previously shown that corpus callosum WM integrity is correlated with unimanual and bimanual task performance in older adults (Fling et al., 2011; Fling and Seidler, 2012). Interestingly, the pegboard and tapping performance measures were not significantly correlated with mean FA in the CST or SLF at study baseline. CST microstructure has been linked to better motor performance in young adults; for example, CST FA increases with motor practice (Reid et al., 2017) and is higher in the hand and arm motor tracts of musicians (Giacosa et al., 2019). It is well established, however, that motor control relies more upon frontal brain activity in older adults than young adults (Seidler et al., 2010; Hawkins et al., 2018). This additional frontal activity during motor task performance in older adults is often interpreted as compensatory (Heuninckx et al., 2008); that is, it is positively associated with better task performance. Davis et al. (2012) have reported that anterior corpus callosum microstructure predicted the strength of functional connectivity between the left and right prefrontal cortex in older adults performing a letter matching task as well as task performance that relied on interhemispheric communication. It is not clear whether the same type of compensation process is taking place in our study, but it is compelling that only FMIN FA was correlated with task performance.

We hypothesized that SLF FA would also correlate with motor performance in our sample, given its potential link to frontal compensation. The SLF connects the frontal, occipital, parietal and temporal lobes (Wang et al., 2016) and SLF FA has been recently shown to predict increasing frailty over a 5 years follow-up (Maltais et al., 2020). It has also been shown to be correlated with tapping speed in individuals with frontotemporal dementia (Henley et al., 2014) and in those who have suffered traumatic brain injury (Farbota et al., 2012). However, the degree of interindividual variance in SLF microstructure in these populations is likely greater than that observed with healthy aging, potentially resulting in the lack of association seen here.

We did not observe any change-change associations in our data; that is, change in WM microstructure was not associated with change in motor performance. Moreover, there were no level-change associations, meaning that average FA at baseline did not predict future motor declines. This is perhaps to be expected given the lack of change over time in CST mean FA and the small slope variance in our measures. Of note, due to the lack of variance in the change trajectories of the motor speed tasks, we could only evaluate longitudinal level-change and change–change associations of WM FA with motor strength in the grip force task. Very recently, grip force has been associated with cognitive decline in healthy aging and demented patients (Cui et al., 2021) and is therefore being discussed also as an early marker of cognitive degradation. While we are still in the beginning of understanding how changes in brain structure impact on changes in motor function, previous longitudinal studies on brain structure and cognitive performance have reported inconsistent results with respect to change-change associations, especially when examining healthy older adults (Salthouse, 2011; Oschwald et al., 2019a).

This is perhaps a reflection of several factors: the good health status and thus high capacity for adaptive compensation for neural decline in this population (Reuter-Lorenz and Park, 2014), the complexity of the cognitive aging process itself, involving a multitude of driving factors (Grady, 2012), and the challenge of modeling the intricate temporal dynamics between two developmental aging processes (King et al., 2018). While the sample that is studied here comprises highly functioning, healthy individuals with expectedly high compensatory ability, they still showed average declines in grip force over the time period studied. These declines, however, are small if compared to previous reports (Auyeung et al., 2014; Patel et al., 2018). Moreover, age-related changes in grip force may be especially multidetermined given its above-mentioned association with cognitive decline (Cui et al., 2021), and its relation to several neural substrates (Carson, 2018). Together, this may serve as an explanation why changes in grip force and FA are not significantly related to each other in our analyses. In addition, while the longitudinal nature of this study is one of its major strength and sets it apart from most of the literature investigating the relations between brain and motor aging, modeling the temporal dynamics between these two developmentally distinct domains still presents a major challenge. In the present study, we used growth modeling to capture simultaneous change-change associations between WM microstructure and motor function. However, more fine-grained temporal investigations of leading-lagging relationships may have been more sensitive to uncover a relationship between these domains (see McArdle et al., 2004; Grimm et al., 2012; Estrada et al., 2019 for such applications in the field of cognitive neuroscience).

In a recent study with the same sample, we reported that changes in SLF FA predict changes in processing speed 2 years later (Oschwald et al., 2019b). It is possible that effects of WM microstructure degradation do not exert immediate effects on motor function, but manifest only after a certain time lag. Unfortunately, the fact that most of the motor function measures in the present study showed only very limited between-person slope variance prohibited reliable modeling of more complex lagged dynamic change processes. However, even in our previous study, only a small subset of the WM tracts investigated (i.e., SLF and anterior thalamic radiation) showed lagged change associations over the studied 4-year interval, which promotes the hypothesis that in the context of healthy aging, with compensation mechanisms being in place, longer time delays might be needed to reveal consistent change-change associations between brain structural and behavior changes.

The high level of health in our participants can also be considered a strength, since many other studies investigate mild cognitive impairment, Alzheimer’s disease patients, or other pathological samples. Understanding the trajectories of neural decline and the related motor impairment in healthy old age is of high practical relevance in our aging society.

To quantify slope variance reliability, we calculated ECR, an index reflecting the slope variance scaled on the effective error and interpretable as a standardized effect size. Effective error variance reflects the size of unsystematic variance in the measure of interest over several measurement occasions. It is influenced by the number of measurement occasions, temporal spacing between these occasions, the overall duration of the study, and the reliability of the measurement instrument itself (Brandmaier et al., 2018). Thus, small slope variance reliability as it was observed for several motor function measures in this study, can reflect a lack of true variance in change and/or low measurement precision that is influenced by a number of study design features. To gain more insights into the longitudinal associations between brain structural and behavior changes, we will have to await future studies that can exploit datasets with more measurement occasions spanning longer time periods. It will be of interest to compute similar measures of slope variance reliability as in this study, to be able to compare these effects across different study designs.

We decided to follow a strictly hypothesis-driven approach in the present study, evaluating only selected WM fiber tracts that have been reportedly involved in motor and cognitive functions relevant for the motor performance tests studied here. It is possible that an analysis approach that evaluates the detailed properties of the tracts (i.e., by running voxel-wise analyses) would have revealed effects that are masked via the averaging of FA values over the entire tract. However, this would have greatly increased the amount of multiple comparisons that would require correcting for, thus substantially lowering power to detect any effects of interest. Furthermore, ROI-based analyses allow the application of sophisticated statistical growth models to our longitudinal data to analyze parallel change processes (which is not feasible in the context of voxel-wise approaches, such as TBSS, which allow only the longitudinal modeling of one change process).

## Conclusion and Future Directions

The current study features the inclusion of multiple longitudinal assessments of both WM microstructure and motor function in a large sample of healthy older adults. Longitudinal assessments across multiple measurement occasions are crucial to better understand trajectories of neural and motor function change in old age and to unravel the effects of central nervous system decline on motor behavior decline over time (Oschwald et al., 2019a). We report evidence for both motor performance and mean WM FA declines over 4 years and negative associations with age. Interestingly, we observed declines in mean FA of the CST only in the older participants but not in the whole sample, which provides support for the “last in, first out” hypothesis of aging which postulates less decline for evolutionarily and developmentally older brain regions and pathways. Mean FA in the FMIN, but not the SLF or CST, correlated with motor speed at baseline. We did not find any longitudinal associations between neural and motor functioning, however. Overall, our results (a) provide important insights into aging-related changes of fine motor abilities and FA in selected WM tracts associated with motor control, (b) support previous cross-sectional work showing that neural control of movement in older adults also involves brain structures outside the core motor system and (c) align with the idea that, in healthy aging, compensatory mechanisms may be in place and longer time delays may be needed to reveal level change or change-change associations. More longitudinal assessments with multiple follow-ups are required to precisely delineate the complex dynamic change associations between neural and motor functioning in aging research.

## Data Availability Statement

The dataset presented in this article is not publicly available because the used consent does not allow for the public sharing of the data.

## Ethics Statement

This study involving human participants was reviewed and approved by the Ethics Committee of the Canton of Zurich. The participants provided their written informed consent to participate in this study.

## Author Contributions

SM and LJ contributed to the design, set-up, maintenance, and support of the Longitudinal Healthy Aging Brain (LHAB) database. JO performed the statistical analysis. JO, RS, and SM wrote the first draft of the manuscript. All authors contributed to manuscript revision, read and approved the submitted version.

## Conflict of Interest

The authors declare that the research was conducted in the absence of any commercial or financial relationships that could be construed as a potential conflict of interest.

## Footnotes

1 In case of the tapping test (left hand), the univariate LGC model did initially converge with a negative slope variance. Constraining the slope variance to a positive value still did not result in an ideal solution, as it produced a warning message (covariance matrix of latent variables non-positive definite). Since these issues were most likely produced by the lack of sufficient slope variance, we added further constraints to the model, fixing the intercept-slope covariance to zero. This resulted in convergence without any warning messages, and a good model fit (see Table 6).
